# Evaluating long-term effectiveness of sleeping sickness control measures in Guinea

**DOI:** 10.1186/s13071-015-1121-x

**Published:** 2015-10-22

**Authors:** Abhishek Pandey, Katherine E. Atkins, Bruno Bucheton, Mamadou Camara, Serap Aksoy, Alison P. Galvani, Martial L. Ndeffo-Mbah

**Affiliations:** Center for Infectious Disease Modeling and Analysis, Yale School of Public Health, New Haven, CT 06510 USA; HAT National Control Program, Ministry of Health, Conakry, Republic of Guinea; UMR INTERTRYP IRD/CIRAD, TA A 17/G, Campus International de Baillarguet, 34398, Montpellier, cedex 5 France; Department of Epidemiology of Microbial Diseases, Yale School of Public Health, New Haven, CT 06510 USA

**Keywords:** Human African trypanosomiasis, Active surveillance, Vector control, Disease elimination, WHO 2020 goals, Tsetse target, Mathematical model

## Abstract

**Background:**

Human African Trypanosomiasis threatens human health across Africa. The subspecies *T.b. gambiense* is responsible for the vast majority of reported HAT cases. Over the past decade, expanded control efforts accomplished a substantial reduction in HAT transmission, spurring the WHO to include Gambian HAT on its roadmap for 2020 elimination. To inform the implementation of this elimination goal, we evaluated the likelihood that current control interventions will achieve the 2020 target in Boffa prefecture in Guinea, which has one of the highest prevalences for HAT in the country, and where vector control measures have been implemented in combination with the traditional screen and treat strategy.

**Methods:**

We developed a three-species mathematical model of HAT and used a Bayesian melding approach to calibrate the model to epidemiological and entomological data from Boffa. From the calibrated model, we generated the probabilistic predictions regarding the likelihood that the current HAT control programs could achieve elimination by 2020 in Boffa.

**Results:**

Our model projections indicate that if annual vector control is implemented in combination with annual or biennial active case detection and treatment, the probability of eliminating HAT as public health problem in Boffa by 2020 is over 90%. Annual implementation of vector control alone has a significant impact but a decreased chance of reaching the objective (77%). However, if the ongoing control efforts are interrupted, HAT will continue to remain a public health problem. In the presence of a non-human animal transmission reservoir, intervention strategies must be maintained at high coverage, even after 2020 elimination, to prevent HAT reemerging as a public health problem.

**Conclusions:**

Complementing active screening and treatment with vector control has the potential to achieve the elimination target before 2020 in the Boffa focus. However, surveillance must continue after elimination to prevent reemergence.

**Electronic supplementary material:**

The online version of this article (doi:10.1186/s13071-015-1121-x) contains supplementary material, which is available to authorized users.

## Background

Human African Trypanosomiasis (HAT) poses a serious health risk to humans in vast regions of Sub-Saharan Africa [1]. Gambian HAT disease progresses over several years from the initial symptoms of fever, headaches and lymphadenopathy (Stage I) through neuropsychiatric disorders and sleep disturbance (hence the name sleeping sickness) (Stage II). If untreated, most HAT cases result in death [1].

The two protozoan subspecies of the parasite *Trypanosoma brucei* (*T.b.*) responsible, *T.b. gambiense* (*Tbg*) and *T.b. rhodesiense* (*Tbr*), are both transmitted by tsetse flies [1, 2]. *Tbg* causes the Gambian form of the disease and is found in 24 countries across West and Central Africa. *Tbg* accounts for over 95 % of HAT cases in Sub-Saharan Africa [3–5]. It is transmitted by the *Palpalis*-group of tsetse, particularly by the subspecies of *Glossina fuscipes* and *Glossina palpalis* [6, 7]. *Palpalis* tsetse are riverine insects and generally infest humid habitats on the fringes of rivers, lake shores and wetlands of West and Central Africa [8, 9]. Due to the absence of a vaccine, intervention strategies against HAT are based on on vector control as well as on “active” and “passive” case detection followed by treatment [7]. Active case detection is implemented by HAT screening of an exposed population, whereas passive case detection relies on self-presentation of HAT patients. After widespread intervention campaigns, the Gambian disease was nearly eradicated in the early 1960s [1, 10]. However, a collapse of surveillance and control activities, often due to periods of political instability, led to disease rebound during the 1990s [11]. With renewed control activities that centered around case detection and treatment, the number of reported cases of Gambian HAT fell by 75 % between 1999 and 2010, from 27,862 to 6,984 [4]. This decline in incidence has spurred the WHO to include Gambian HAT on its 2020 roadmap for the elimination of neglected tropical diseases [5]. The WHO 2020 HAT elimination goal seeks to eliminate Gambian HAT as a public health problem by reducing the annual incidence rate to less than 1 in 10,000 people in 90 % of endemic foci [5]. It is unclear if the WHO HAT elimination goal is feasible under current interventions or if complementary strategies will have to be considered.

We developed a mathematical model for HAT to assess the likelihood that ongoing vector control, active screening and treatment strategies could eliminate Gambian HAT as a public health problem by 2020. We focus on the Boffa prefecture in Guinea as our study area, which has one of the highest prevalence for HAT in Guinea [12–14]. We fitted our model to HAT infection prevalence between 2008 and 2013 in Boffa East using a Bayesian inference approach to capture uncertainty of epidemiological data into model projections. We used scenario analysis to evaluate the robustness of our predictions to the existence of a non-human animal transmission reservoir.

## Methods

### Model

**Fig. 1:**
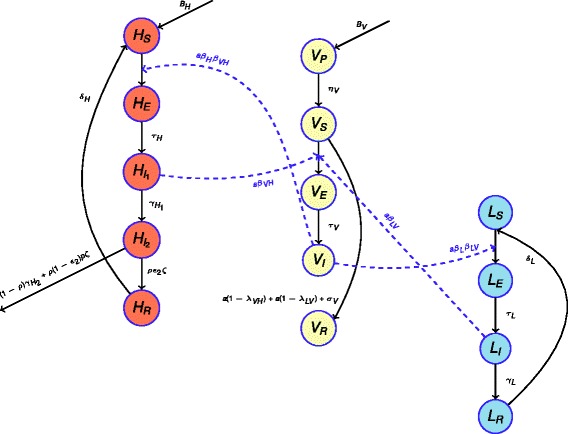
Model diagram of epidemiological compartments (circles) with rates of movement between each compartment (arrows). Further details are presented in the Methods

Gambian HAT is generally regarded as a disease primarily infecting humans, with uncertainty about the role of non-human animals in sustaining the transmission cycle. We developed two mathematical models, with and without a non-human animal transmission reservoir. Here, we describe the model that includes the non-human reservoir reservoir. To evaluate the long-term effectiveness of HAT control measures in Guinea, we developed a three-species SEIR differential-equation model for *T.b. gambiense* infection among tsetse (*V*), humans (*H*) and non-human animals (NHA) (*L*) based on previous mathematical models [15, 16]. Each species was categorized in terms of their infection status: susceptible (*V*_*S*_, *H*_*S*_, *L*_*S*_), exposed (*V*_*E*_, *H*_*E*_, *L*_*E*_), infectious (*V*_*I*_, *H*_*I*_, *L*_*I*_) or recovered (*V*_*R*_, *H*_*R*_, *L*_*R*_) (Fig. 1, Additional file 1). To capture the reality that tsetse flies are more likely to become infected during their first bloodmeal and the susceptibility of tsetse to trypanosome decreases with age (hours after eclosion of the fly from the puparium) at first meal [17], we assumed that tsetse are susceptible to trypanosome infection only during their first blood-meal and within 24 h after emergence from pupa (*V*_*P*_) to the adult stage (*V*_*S*_) [17]. Susceptible tsetse (*V*_*S*_) become infected after feeding on an infectious human or NHA and enter the exposed state (*V*_*E*_) during which the infection incubates. After incubation, tsetse become infectious (*V*_*I*_) for the rest of their life and can transmit infection to human and non-human animals. We assumed that the tsetse population has a density-dependent mortality rate:

1 $$ {\mu}_V={\mu}_{V_0}\left(1+{\mu}_{V_1}V\right), $$

where ${\mu}_{V_0}$ is the death rate in the absence of intra-species competition, ${\mu}_{V_1}$ measures the effect of intra-species competition on death rate, and *V* is the population size of all non-pupal tsetse.

Both humans and NHAs may become exposed to infection after being bitten by an infectious tsetse (*V*_*I*_). After the incubation period, humans and NHA hosts enter the infectious stage of HAT (${H}_{I_1}$ and *L*_*I*_ respectively), in which they can transmit infection to susceptible tsetse if bitten. Human hosts progress to stage II of infection $\left({H}_{I_2}\right)$, in which they are assumed not to be infectious due to isolation arising from the severity of symptoms in this stage of disease progression [1, 18]. Infected people in stage II either seek treatment and recover (*H*_*R*_) or die due to being untreated or due to treatment failure. Successfully treated HAT patients are temporarily resistant to reinfection and exposure via tsetse (through immunity to reinfection or hospitalization, respectively) before returning to full susceptibility (*H*_*S*_). Infectious NHAs (*L*_*I*_) clear infection and become temporarily immune to reinfection (*L*_*R*_) before returning to the susceptible state (*L*_*S*_). We assumed that both human and NHA populations are of constant size throughout the analysis.

Our model captured the annual active screening and treatment in Boffa, by assuming that active surveillance occurs within a 20 day-period, during which a proportion, *ϕ*, of the human population is tested for *Tbg* using the Card Agglutination Test for Trypanosomiasis (CATT) and then a Trypanolysis test (TL). While the CATT diagnostic tool can be performed *in situ* using blood from a finger prick, TL is performed in laboratories using plasma samples [19]. TL has been implemented successfully in Guinea, Cote d’Ivoire and Burkina Faso during medical surveys with a 100 % specificity, compared to 91 % for CATT [20]. Therefore the combined CATT-TL testing has a sensitivity of *ρ* and specificity of 100 %. Once infection status has been confirmed, a lumbar puncture is performed to determine whether the patient has progressed to stage II. We assumed that all patients testing positive were treated [21], such that a fraction, *ϕρϵ*_1_ and *ϕρϵ*_2_, of stage I and stage II HAT patients recover after 20 days of active surveillance (where *ϵ*_1_ and *ϵ*_2_ are the efficacies of stage I and stage II treatment).

To reduce transmission, insecticide-treated targets have been deployed in areas where people live and work [21]. Consistent with previous model calibration work [22] that showed temporary efficacy of this type of vector control, we assumed that for the initial three months of deployment, the targets induced a tsetse maximal kill rate of *x*, which decreased linearly to zero in the following three months, and remained ineffective for the subsequent six months until the targets were replaced [22].

### Data sources

To calibrate and validate our dynamic model, we used multiple data sources from the Boffa region of Guinea. For calibration, we used four data sources: i) the number of HAT cases (partitioned by stage) diagnosed and treated in the Boffa East region through active annual screening activities in 2008 and 2013 with known coverage [12, 21], ii) the *Tbg* prevalence in non-human animal reservoir across Boffa East, iii) the *Tbg* prevalence in tsetse across Boffa East, and iv) the reduction in tsetse density between 2012 and 2013 in Boffa East as a result of a vector control program. For validation, we used i) the number of HAT cases (partitioned by stage) diagnosed and treated in Boffa East through active annual screening activities in 2010 and 2012 [12, 21], and ii) the HAT incidence in 2012 [21]. To parameterize human population size, we used data from a 2011 census of Boffa. As previous a study has shown *Tbg* prevalence to be extremely low in tsetse and NHA [12], we assumed that both of these prevalences were under 1 %. All other parameters were based on published estimates (Table 2).

### Model fitting

**Fig. 2:**
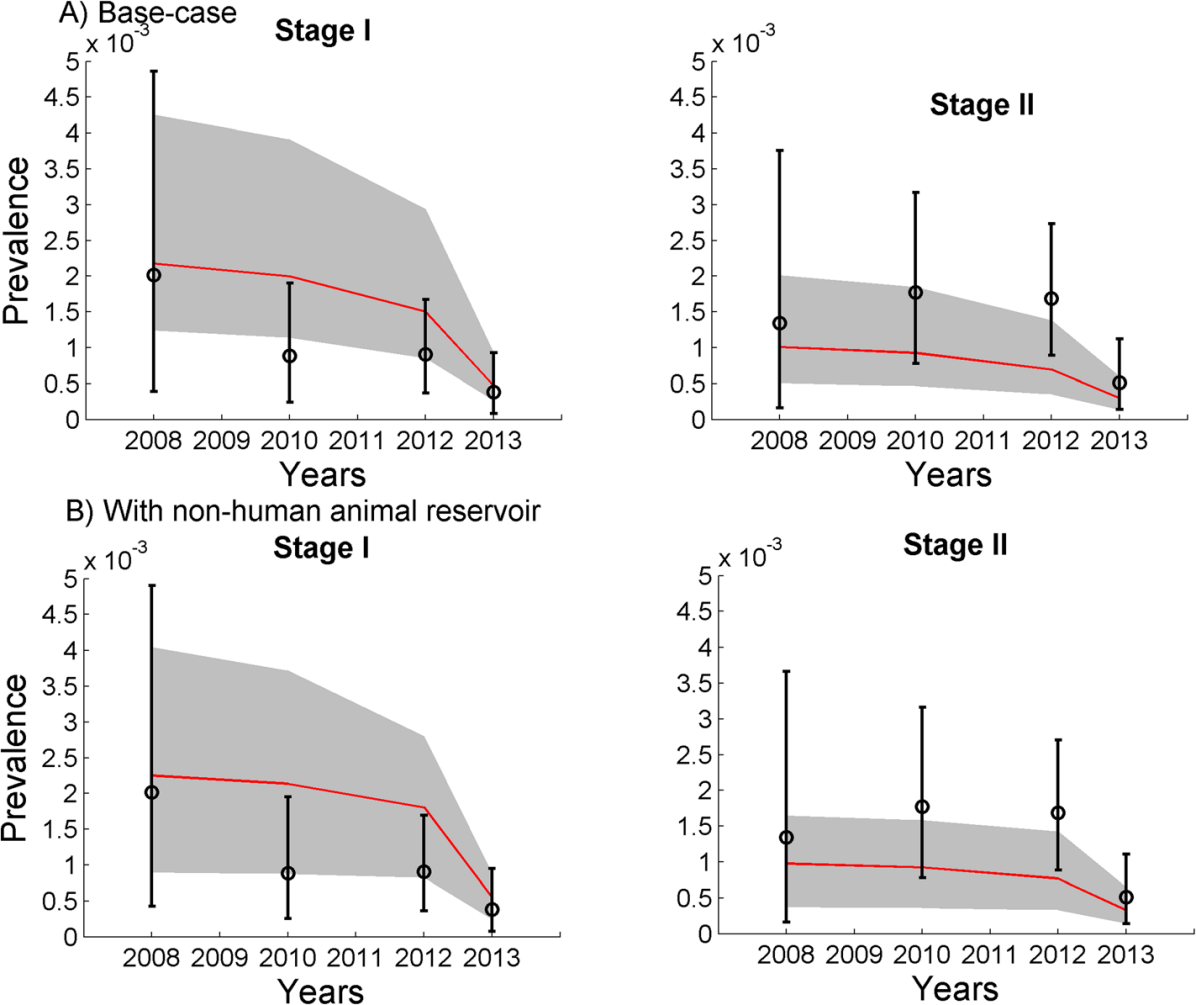
Model fits (**a**) base-case model without a non-human animal (NHA) reservoir, and (**b**) model with an NHA reservoir. Trajectories of the model fitted to prevalence data for stage I and stage II HAT cases from Boffa East mainland using Bayesian melding for years 2008 and 2013 and validated using 2010 and 2012. The red lines represent the HAT phase I and phase II prevalence estimated by our model for baseline epidemiological parameters (the grey areas represent the 95 % confidence intervals)

To estimate posterior distributions for the unknown epidemiological parameters—probability of tsetse bite on humans (*β*_*VH*_), transmission probability from tsetse to humans (*β*_*H*_), treatment seeking rate of stage II patients (*ζ*) and transmission probability from tsetse to NHA (*β*_*L*_)—we used a Bayesian melding method [23, 24] to calibrate the model to prevalence data for stage I and stage II HAT cases in Boffa East Mainland in 2008, as well as Trypanosomiasis prevalence among tsetse and the NHA reservoir [12, 21]. Given that there was no active screening or vector control in Boffa between 2000 and 2008, we assumed an equilibrium prevalence prior to 2008. We modeled the active screening with case treatment in 2008, 2010, and 2012 across Boffa East with the observed 10.2 %, 31.2 %, and 53.4 % coverage respectively. The deployment of vector control through insecticide-treated targets in 2012 decreased tsetse density by 60 % over a year [21]. To estimate the maximal kill rate, *x*, we used maximum likelihood estimation with normal distribution as the likelihood function to fit the model to this reduction in tsetse density [21]. The model was run until 2013 for best fit parameters from Bayesian fitting with active screening and treatment in year 2008, 2010, 2012 followed by vector control in year 2012. We refined the posterior distributions of the epidemiological parameters (*β*_*VH*_, *β*_*H*_, *ζ* , *β*_*L*_), obtained from the initial Bayesian fitting of the model to the 2008 data which were then used as prior distributions for fitting the model to 2013 data of HAT stage I and II prevalences (Fig. 2).

The Bayesian melding method takes all available prior information on model inputs and, when combined with a likelihood function, generates posterior distributions of model parameters and predicted model outputs through statistical comparisons of model predictions with observed data. We used a uniform prior distribution for each model parameter (*β*_*VH*_, *β*_*H*_, *ζ*,  *β*_*L*_) and Beta distributions for the observed data on stage I and stage II HAT, tsetse and NHA trypanosomiasis prevalence. To describe the Bayesian melding algorithm, we denote the simulation model (Additional file 1) by *M*, the epidemiological parameters (*β*_*VH*_, *β*_*H*_, *ζ*,  *β*_*L*_) as *Θ* and the model-predicted output as *Γ* = *M*(*Θ*). We denote the prior distribution for each model parameter as *q*(*Θ*). We denote the data as *W* and the associated likelihood of the model outputs as *L*(*Γ*) = Pr (*Γ*|*W*). The posterior distribution of inputs is then proportional to *q*(*Θ*)*L*(*Γ*). We implemented a sample-importance-resample algorithm to approximate the posterior distribution. First, we generated a set of input parameters, *Θ*(*i*), by randomly sampling from the respective prior distributions *i* times. We then evaluated the model using that set of parameters, *Γ*(*i*) = *M*(*Θ*(*i*)) for each run *i*. Next, we calculated the corresponding likelihood for the model run. For each sample *Θ*(*j*), with non-zero corresponding likelihood, the sampling weight was $\varOmega (j)=L\left(\varGamma (j)\right)/{\displaystyle \sum_{k=1}^{Nl}}L\left(\varGamma (k)\right)$. To ensure a sufficient sample from the posterior distributions, we set *i* = 400,000.

The number of non-zero likelihood samples, *N*_*l*_, was 520 for the model without NHA reservoir, and 1,053 for the model with an NHA when fit to 2008 prevalence data. After refinement to fit the models to 2013 data, the final sample size was 290 for the model without an NHA reservoir, and 395 with an NHA reservoir. We repeated this sampling procedure 10,000 times with replacement, using a probability of selection proportional to the sampling weights to obtain an approximation of the posterior distribution for the inputs. Output from the simulation resampled most frequently (i.e., the simulation most compatible with empirical prevalence data) represents the estimated mode for the output parameters of interest. The 2.5th and 97.5th percentiles of the inputs (and corresponding model outputs) correspond to 95 % credible limits.

### Effectiveness of intervention strategies

We evaluated the disease dynamics under two model structures: first, with no NHA reservoir, following a survey in Boffa showing no evidence of NHA infection [12], and second, with a small prevalence of NHA infection, following studies suggesting the importance of NHAs in the maintenance of disease transmission [25]. Using the calibrated models, we projected the model between 2013 and 2030 under the assumption that all control measures are implemented with the same coverage and efficacies as achieved in year 2012, with the exception of years 2014 and 2015 when no control measures were implemented due to the Ebola crisis. We then evaluated the probability of reaching the WHO elimination threshold of less than one case per 10,000 annually, which corresponds to a “low transmission focus”, and also the more stringent condition of 1 case per 100,000 inhabitants, which corresponds to a “very low transmission focus” [5]. We assessed the probability of elimination under three different control interventions: annual vector control effort, annual vector control effort with annual active-screening and treatment, and annual vector control effort with biennial active-screening and treatment.

### Model code

The analysis was performed using Matlab R2014b, and all model codes are available via https://github.com/abhiganit/Sleeping-Sickness.

## Results

We employed a Bayesian melding approach to fit our models, with and without NHA reservoir, to epidemiological data of HAT in Boffa East, Guinea. Using prior distributions of epidemiological parameters based on estimates available in the literature (Table 1), we derived a posterior distribution for each epidemiological parameter for which the model gives the best estimate for stage I and stage II HAT prevalences in 2008 and 2013 and trypanosomiasis prevalence among tsetse and NHA reservoir (Fig. 2, Tables 2 and 3). The prevalence of stage I and Stage II HAT in 2010 and 2012 and the stage I incidence in 2013 were used for model validation (Fig. 2 and Table 3).

**Table 1:** Definitions and values for model parameters

Parameter	Definition	Value	Reference
V/H	Number of tsetse flies (V) per human (H)	17	[15]
L/H	Number of NHA (L) per human (H)	1/6	[15]
H	Population size of Boffa East Mainland in 2008	14,500	Unpublished data
*B*_*V*_	Tsetse constant birth rate	0.05/day	[15]
1/*η*_*V*_	Duration of pupae stage in tsetse	20 days	[18]
${\mu}_{V_0}$	Tsetse death rate without competition	0.030/day	[15]
${\mu}_{V_1}$	Death rate competition parameter	0.0002	Assumed
1/*σ*_*V*_	Susceptibility period in tsetse	1 day	[15]
*a*	Tsetse biting rate	0.333/day	[15]
*β*_*VH*_	Probability of tsetse bite on human	See Table 2	Estimated
*β*_*VL*_	Probability of tsetse bite on NHA	min(1-*β*_*VH*_,0.71)	[25]
1/*τ*_*V*_	Incubation period in tsetse	25 days	[15]
*μ*_*H*_	Human constant death rate	4.66e-05 /day	[34]
*β*_*H*_	Transmission probability from tsetse to humans	See Table 2	Estimated
1/*τ*_*H*_	Incubation period in humans	12 days	[15]
1/*γ*_*H*1_	Stage I infectious period without treatment	526 days	[35]
1/*γ*_*H*2_	Stage II infectious period without treatment	252 days	[36]
1/*δ*_*H*_	Immune period in humans after treatment	50 days	[15]
*β*_*L*_	Transmission probability from tsetse to NHA	See Table 2	Estimated
*β*_*V*_	Transmission probability from humans/NHA to tsetse	0.2	[2, 15]
1/*τ*_*L*_	Incubation period in NHA	12 days	[15]
1/*γ*_*L*_	Infectious period in NHA	50 days	[15]
1/*δ*_*L*_	Immune period in NHA	50 days	[15]
*ϕ*	Coverage of active surveillance	Varies	-
*ρ*	Probability that a HAT patients gets a positive CATT and then a positive antibody/Trypanolysis test	0.87	[1]
*ε*_1_	Efficacy of stage I treatment (pentamidine)	0.94	[37]
*ε*_2_	Efficacy of stage II treatment (nifurtimox-eflornithine)	0.965	[38]
*ζ*	Treatment seeking rate of stage II patients	See Table 2	Estimated
*p*	Probability of death due to stage II treatment failure (nifurtimox-eflornithine)	0.007	[38]

**Table 2:** Prior and posterior distribution of parameter estimates^a^

No non-human animal reservoir (Base-case)		
Parameter	Prior	Median (95 % CI)
*β*_*VH*_	U(0.1,0.6)	0.3750 (0.1698, 0.5626)
*β*_*H*_	U(0,1)	0.1751 (0.0778,0.8550)
1/*ζ*	U(0,252)	202 (97,250)
*x*	-	0.0503
With non-human animal reservoir		
Parameter	Prior	Median (95 % CI)
*β*_*VH*_	U(0.1,0.6)	0.3020 (0.1021, 0.5932)
*β*_*H*_	U(0,1)	0.0076 (0.0011,0.3265)
1/*ζ*	U(0,252)	203 (101, 243)
*β*_*L*_	U(0,1)	0.1345 (0.0476, 0.6619)
*x*	-	0.0503

^a^Likelihood function was formed using beta distributions for stage I and stage II HAT cases in year 2008 (Stage I: Beta(3,1488), Stage II: Beta(2,1488) and in year 2013 (Stage I: Beta(3,7788), Stage II: Beta(4,7788)

**Table 3:** Model calibration to tsetse and NHA trypanosome prevalence and validation to 2013 stage I HAT incidence

	Data	Estimates from base-case model (no NHA reservoir)	Estimates from model with NHA
Tsetse prevalence (2008)	Assumed < 1 %	0.0018 % (95 % CI:0.001–0.0040 %)	0.08 % (95 % CI:0.002–0.4 %)
NHA prevalence (2008)	Assumed < 1 %	Fixed at 0 %	0.3 % (95 % CI: 0.0005–0.9 %)
Stage I HAT incidence (2013)	0.07 % (95 % CI: 0.01–0.2 %)	0.15 % (95 % CI: 0.07–0.2 %)	0.17 % (95 % CI: 0.06–0.21 %)

**Fig. 3:**
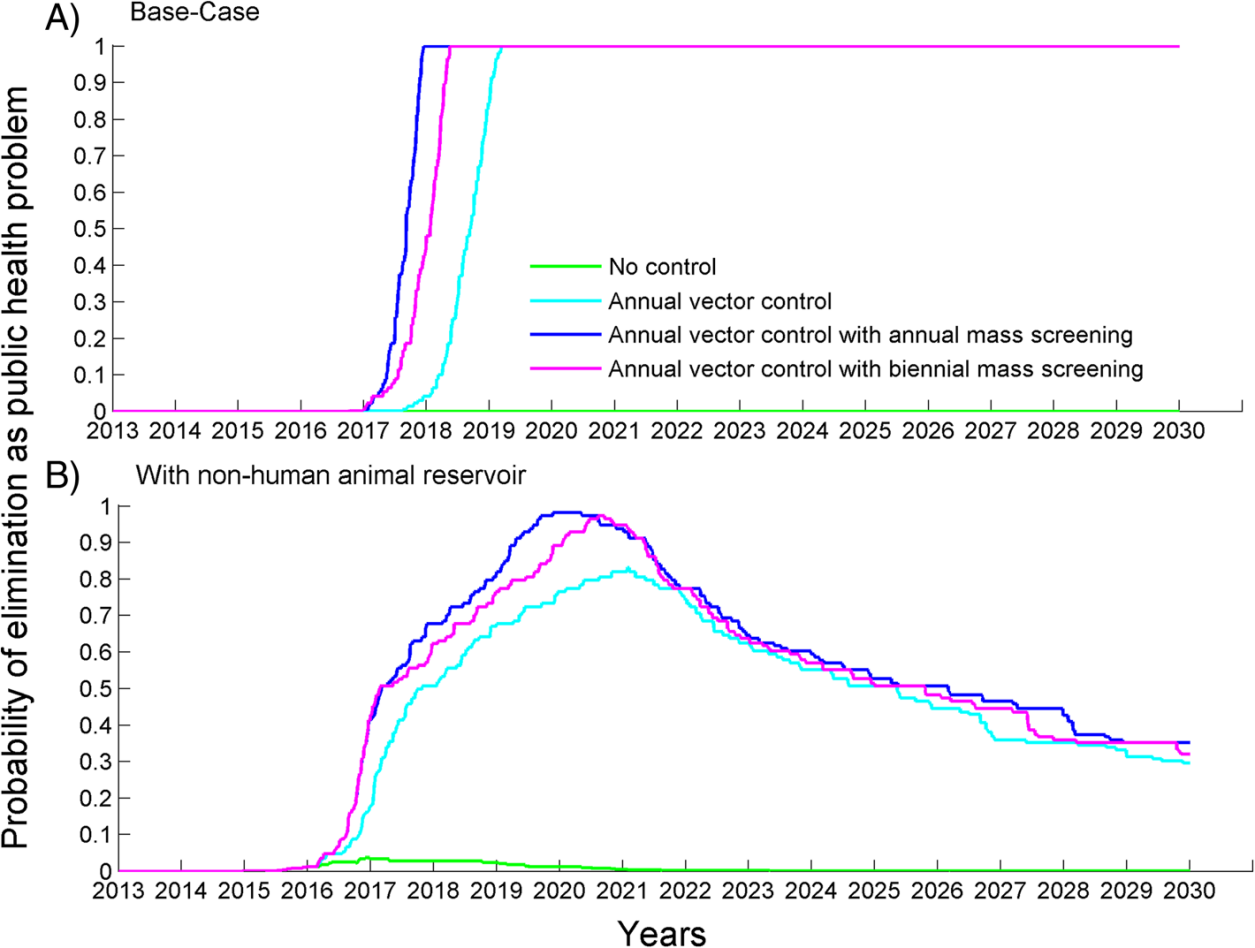
Probability of HAT elimination as public health problem under various control strategies (**a**) in absence of a non-human animal (NHA) reservoir, and (**b**) with an NHA reservoir. Vector control and active screening and treatment are implemented with the 2012 efficacy and coverage

We projected the trajectory of our model until 2030 and estimated the probability of HAT elimination as a public health problem by 2020 under various control interventions (Fig. 3). Predictions from our model indicate that if no further control intervention is implemented after 2015, HAT will likely continue as a public health problem in Boffa East with annual incidence exceeding 2 cases per 10,000 individuals by the end of 2020. To evaluate the likelihood of achieving the WHO 2020 goal of HAT elimination as a public health problem in Boffa, we assumed that the intervention strategies are continuously implemented from 2016 until 2020, at which time control efforts are ceased. Our base-case model, without NHA reservoir, showed that annual implementation of vector control at the same efficacy as 2012 is likely to eliminate HAT as a public health problem by 2020, with the new cases remaining less 1 per 10,000 people (Fig. 3a). If vector control is complemented by annual active screening and treatment, then the model predicts that a 100 % probability of elimination is achieved in 2018. With only biennial implementation of active screening and treatment, elimination is achieved in 2019 (Fig. 3a).

When we incorporated a non-human animal transmission reservoir, our model predicts at least 77 % probability of HAT elimination as a public health problem in Boffa East by 2020 under the three control strategies if the coverage and efficacy remains consistent (Fig. 3b). Though likelihood of elimination is high, none of the three control strategies implemented at their 2012 efficacy level guarantee elimination of HAT as it was the case in the absence NHA reservoir (compare Fig. 3a to b). Moreover, in the presence of an NHA reservoir, intervention strategies must be maintained at high coverage, even after 2020 elimination, to prevent HAT reemerging as a public health problem in Boffa East by 2025 (Fig. 3b).

**Fig. 4:**
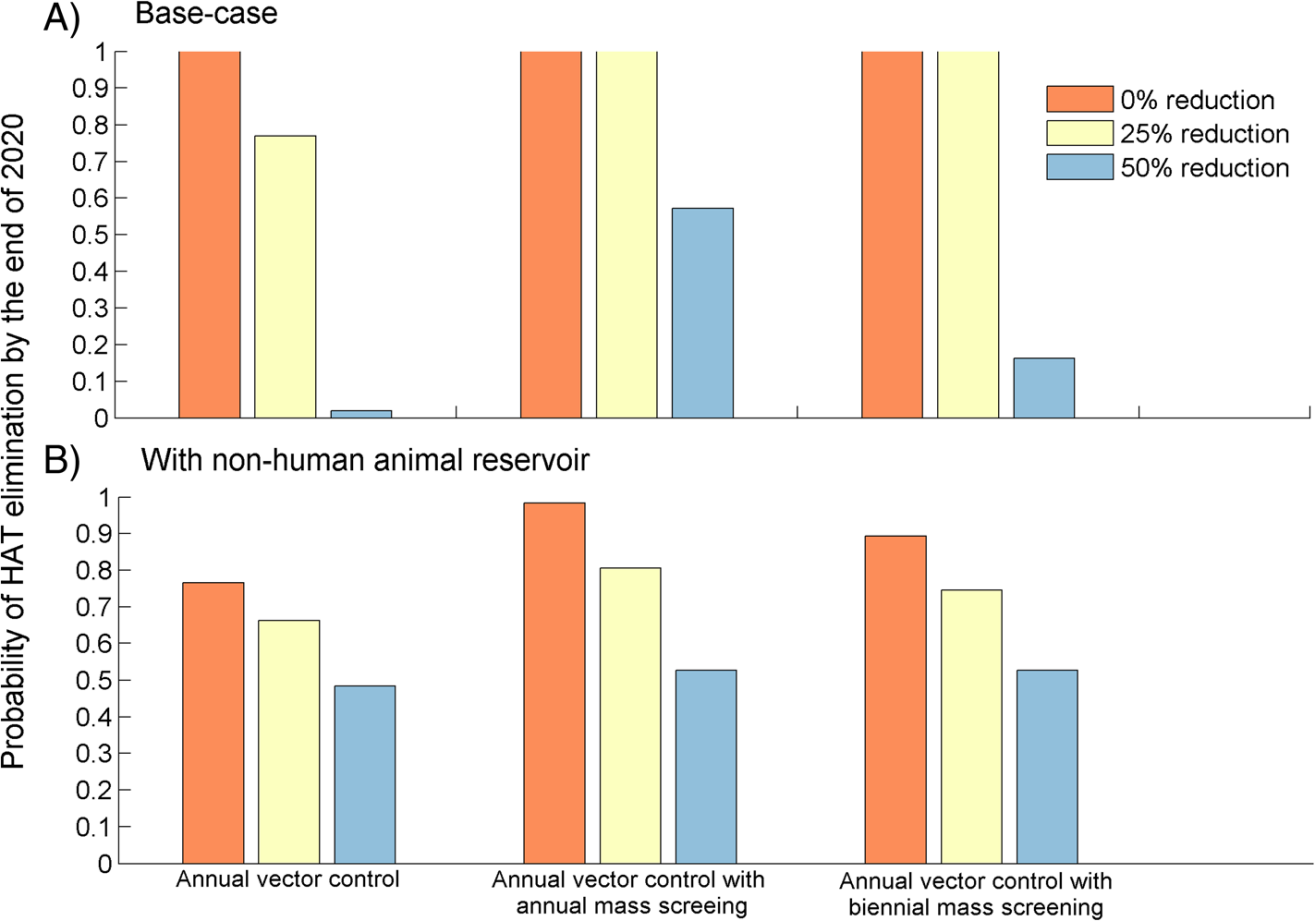
Probabilities of HAT elimination as public health problem by the end of 2020 (a) in the absence of a non-human animal (NHA) reservoir, and (b) with an NHA reservoir. All controls were implemented either annually or biennially and different colors represent different reduction levels of vector control efficacy and active screening coverage relative to 2012 efficacy and coverage

Maintaining a high efficacy of vector control, high density of insecticide-treated targets, may be unsustainable in the long term. Our results indicate that if vector control efficacy is reduced by 25 %, this would lower the probability of HAT elimination as a public health problem by the end of 2020 to 77 %. Moreover, a 50 % reduction in vector control efficacy would likely prevent elimination by the target date, with only a 1.9 % probability of attaining the goal (Fig. 4a).

In the presence of NHA reservoir, a reduction of vector control efficacy by 25 % would lower the probability of HAT elimination as a public health problem by the end of 2020 from 77 to 67 % (Fig. 4b). A 50 % reduction in efficacy would lower the probability of HAT elimination to 49 % (Fig. 4b). If vector control is combined with active screening and treatment, a 50 % reduction in vector control efficacy and active screening coverage achieves a 53 % probability of HAT elimination by 2020 for annual and biennial active screening, compared with 16 % for biennial and 57 % for annual active screening in the absence of an NHA reservoir (Fig. 4).

**Fig. 5:**
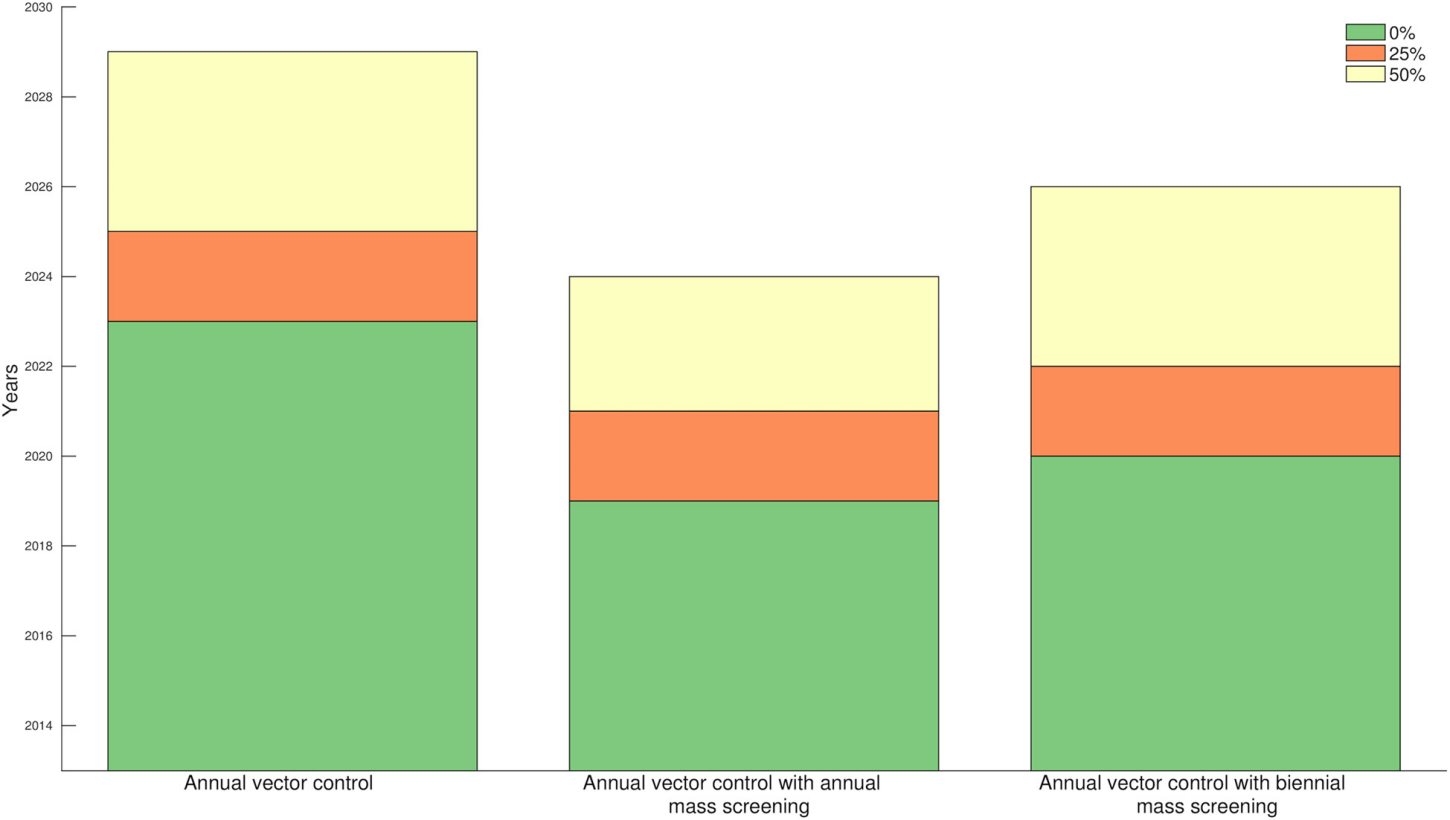
Years to elimination with 100% probability under threshold of less than 1 new case per 100,000 people for the different control strategies. Different colors represent different proportional reduction of vector control efficacy and active screening coverage relative to 2012 efficacy and coverage

Considering that current control efforts are predicted to be sufficiently efficacious to meet the WHO HAT elimination goal in Boffa East Mainland, we evaluated time to elimination under the more stringent criteria of 1 cases per 100,000 people when control interventions are continuously implemented until 2030. We found that vector control implemented annually will meet the threshold if it is continued through 2023. If the annual vector control is complemented with annual or biennial active screening, the threshold can be achieved by the end of 2020 and 2021, respectively (Fig. 5). We also found that if the controls are implemented with lower efficacy and coverage, the control will have to be continued at least until 2025 to meet the threshold (Fig. 5).

We used *Akaike Information Criterion* (AIC) to compare our base-case model and the model with an NHA reservoir. The model without NHA had smaller AIC. However, the relative likelihood for the model with NHA was 27 % and the difference in AIC value between the models was 2.64, suggesting that both models are plausible [26]. Thus, while there is some support for preferring the simpler model that includes an NHA reservoir, there is limited evidence for either the presence of absence of an NHA transmission reservoir.

## Discussion

We developed a mathematical model of HAT transmission calibrated to data from Boffa East in Guinea to evaluate the effectiveness of vector control combined with active screening to eliminate HAT as a public health problem in this disease focus. Annual vector control combined with annual or biennial active screening and treatment is predicted to achieve the WHO HAT elimination goal by 2020 in Boffa East, if maintained at the current efficacy. Vector control alone leads to at least a 77 % probability of achieving the elimination goal. If current control efficacies cannot be sustained, lower probabilities of elimination by 2020 are predicted. In the presence of a non-human animal reservoir, our model predicts an increased risk of HAT reemerging as a public health problem in Boffa East after 2025, if control efforts are not continued after the public health elimination goal is achieved in 2020.

While active screening and treatment has effectively controlled HAT in many foci [27], some areas that have not concomitantly adopted vector control have failed to bring HAT under control [7]. There is also evidence to suggest that there are asymptomatic carriers and seropositive cases who are not detected by parasitological techniques, which will likely limit the effectiveness of active screening and treatment for breaking the HAT transmission cycle [25, 28, 29].

Consistent with a mathematical model fitted to data from the Democratic Republic of Congo [39], our model does not inform the question of whether there exists a non-human animal reservoir. However, the existence of a NHA reservoir impacts the chance of sustaining HAT elimination. We found that, in the presence of a non-human animal reservoir, HAT was more resistant to elimination, with a heightened risk of becoming a public health problem after 2020 again if control efforts are interrupted. Moreover, when there is a NHA transmission reservoir, using vector control strategies to complement HAT treatment programs is crucial to achieve elimination.

The impact of control strategies in disease foci depends on the trypanosomiasis prevalence among tsetse and NHA reservoir. As the tsetse and NHA prevalence in Boffa is relatively low [12], it may take longer to reach elimination in higher intensity areas. For example,  Rock et al. [39] showed that HAT elimination in the high endemicity region of Democratic Republic of Congo is highly unlikely to be reached by 2020 with current controls. Regardless, control efforts should be sustained following HAT elimination as a public health problem, as the possibility of a NHA reservoir poses a risk for reemergence, as would transmission from surrounding regions. Although we assumed specific ratios of human to tsetse and humans to NHA different ratios would have a marginal impact on our results, as these variations will be absorbed into the transmission parameters that were fitted to prevalence data.

The data from medical surveys conducted in Guinea use the Trypanolysis test (TL) to ensure accuracy of HAT patient reporting. However, many countries rely on CATT and, if positive, a parasitology test for confirmation. As CATT has a relatively low specificity and a parasitology test has a relatively low sensitivity, many foci will suffer from under- or over-diagnosis, respectively. Such misdiagnosis will implicitly lower or raise the stringency of the elimination threshold, respectively.

Previous studies have suggested that tsetse may bite a host more than once per feeding cycle, shortening the feeding interval of tsetse flies to less than three days [30, 31]. A shorter cycle increases the biting rate of tsetse, resulting in a higher probability that an infected tsetse fly will infect a susceptible host, and thus increasing the effectiveness of vector control.

In our study, we assumed that tsetse feed randomly on individuals in a given species, such that individuals are equally likely to be bitten by tsetse, and thereby exposed to trypanosomiasis. However, empirical studies have observed heterogeneity in exposure to tsetse bites within humans and livestock [21, 32]. Within-species heterogeneity suggests that either tsetse flies may be preferentially feeding on certain individuals within a given species or that some individuals are geographically more exposed to tsetse habitats. Notwithstanding variation in tsetse bite exposure, some individuals may be more genetically susceptible to HAT infection [28], which could impact elimination goals. For example, a reservoir of undiagnosed cases who maintain a transmission reservoir will truncate the effectiveness of case finding and treatment. Similarly, clustering of incidence in remote sub-populations will also limit the effectiveness of human-targeted control interventions. Heterogeneities in tsetse exposure would be important to explore in future work assessing the sustainability of HAT elimination [21].

Given that tsetse population sizes fluctuate seasonally and vary between foci [15], human exposure to tsetse and therefore HAT transmission dynamics may exhibit seasonal and geographical variation [33]. Our model accounts for the impact of seasonal variation on the efficacy of insecticide-treated target traps using a relatively simple step function to capture the decrease in traps efficacy over time from seasonal and non-seasonal factors. With data from enhanced surveillance techniques across wider areas, future modeling may be able to explicitly incorporate seasonality in tsetse population dynamics and human–tsetse contact.

## Conclusion

We evaluated the feasibility of HAT elimination goal by 2020 in Boffa East focus using a data-driven mathematical model by calculating probabilistic estimates of whether the current HAT control program is likely to achieve HAT elimination as a public health problem by 2020. Our analysis revealed that combinations of annual vector control with active-screening will most likely meet the WHO 2020 elimination goal. Furthermore, the combined control implemented with the coverage and efficacy achieved in 2012 is predicted to be sufficient to reduce incidence below 1 case per 100,000 people by 2020. As vector control works synergistically with active screening and treatment, using a combined approach for Boffa has the potential to meet the WHO goal by 2020 in Boffa focus.

## Additional file

Additional file 1:
**Model Equations.** (PDF 73 kb)
