# Supplementary material for: Evaluating long-term effectiveness of sleeping sickness control measures in Guinea
Source: Parasit Vectors. 2015 Oct 22;8:550. doi: 10.1186/s13071-015-1121-x (PMC4618537; doi:10.1186/s13071-015-1121-x)
Supplement: Additional file 1: — Model Equations. (PDF 73 kb) [file 13071_2015_1121_MOESM1_ESM.pdf]

Model Equations:

$$
\begin{aligned}
\frac{dV_P}{dt} &= B_V V - \eta_V V_P \\
\frac{dV_S}{dt} &= \eta_V V_P - (a + \sigma_V + \mu_V + x)V_S, \\
\frac{dV_E}{dt} &= (a\lambda_{VH} + a\lambda_{VL})V_S - (\tau_V + \mu_V + x)V_E, \\
\frac{dV_I}{dt} &= \tau_V V_E - (\mu_V + x)V_I, \\
\frac{dV_R}{dt} &= [a(1 - \lambda_{VH}) + a(1 - \lambda_{VL}) + \sigma_V]V_S - (\mu_V + x)V_R, \\
\frac{dH_S}{dt} &= B_H + \delta_H H_R - a\beta_{VH}\beta_H V_I \frac{H_S}{H} - \mu_H H_S, \\
\frac{dH_E}{dt} &= a\beta_{VH}\beta_H V_I \frac{H_S}{H} - (\tau_H + \mu_H)H_E, \\
\frac{dH_{I_1}}{dt} &= \tau_H H_E - (\gamma_{H_1} + \mu_H)H_{I_1}, \\
\frac{dH_{I_2}}{dt} &= \gamma_{H_1} H_{I_1} - [\rho\epsilon_2\zeta + (1 - \rho)\gamma_{H_2} + \rho(1 - \epsilon_2)p\zeta + \mu_H]H_{I_2}, \\
\frac{dH_R}{dt} &= \rho\epsilon\zeta H_{I_2} - (\delta_H + \mu_H)H_R, \\
\frac{dL_S}{dt} &= \delta_L L_R - a\beta_{VL}\beta_L V_I \frac{L_S}{L}, \\
\frac{dL_E}{dt} &= a\beta_{VL}\beta_L V_I \frac{L_S}{L} - \tau_L L_E, \\
\frac{dL_I}{dt} &= \tau_L L_E - \gamma_L L_I, \\
\frac{dL_R}{dt} &= \gamma_L L_I - \delta_L L_R.
\end{aligned}
$$

where human birth rate $B_H = \mu_H H + [(1 - \rho)\gamma_{H_2} + \rho(1 - \epsilon_2)p\zeta]H_{I_2}$, the tsetse density-dependent mortality rate $\mu_V = \mu_{V_0}(1 + \mu_{V_1} V)$ and the force of infections

$$
\begin{aligned}
\lambda_{VH} &= \beta_V \beta_{VH} \frac{H_{I_1}}{H}, \\
\lambda_{VL} &= \beta_V \beta_{VL} \frac{L_I}{L}.
\end{aligned}
$$
